# Evaluation of the Prognostic Value of CD56 (140 kDa Isoform) Expression in Breast Cancer Tissues: an Eight-Year Retrospective Study

**DOI:** 10.52547/ibj.26.3.175

**Published:** 2022-04-03

**Authors:** Kianoush Niknam, Akbar Safaei, Abbas Ghaderi

**Affiliations:** 1 Shiraz Institute for Cancer Research, School of Medicine, Shiraz University of Medical Sciences, Shiraz, Iran;; 2 Department of Pathology, School of Medicine, Shiraz University of Medical Sciences, Shiraz, Iran;; 3 Department of Immunology, School of Medicine, Shiraz University of Medical Sciences, Shiraz, Iran

**Keywords:** Breast cancer, Neural cell adhesion molecule, Prognosis

## Abstract

**Background:**

Identification of specific antigens is highly beneficial for early detection, diagnosis, staging, and outcome prediction of cancer. This study aimed to evaluate the expression and prognostic value of CD56 (140 kDa isoform) in IDC.

**Methods:**

Sixty-five patients with IDC who underwent radical surgery or mastectomy as the primary treatment were included. Proper formalin-fixed and paraffin embedded tissue blocks of the patients were prepared and stained by IHC for CD56 (140 kDa isoform) molecule. Chi-square and fisher exact tests were used to compare the results against the clinicopathologic data of patients. Kaplan-Meier and log-rank test were employed to study the prognostic value of the target antigen.

**Results:**

The expression pattern of CD56 was granular and cytoplasmic. There were significant associations between the intensity of CD56 expression in invasive cells and carcinoma *in situ* (*p* = 0.005) and normal ducts (*p* = 0.010). Among all clinicipathologic parameters, there was only a significant association between the expression of ER and CD56 (*p* = 0.023). Neither OS (*p* = 0.356) nor DFS (*p* = 0.976) had significant correlation with CD56 expression.

**Conclusion:**

Our data indicated that the CD56 marker offers no prognostic value in terms of predicting the OS or DFS for up to eight years after primary surgery. Furthermore, the intensity of its expression is similar between normal, non-invasive, and invasive cells. Considering the generally better outcome of ER+ BC patients than their ER-counterparts, the CD56 marker may be indirectly associated with a more favorable prognosis among IDC patients.

## INTRODUCTION

Breast cancer is one of the most prevalent malignancies among females. Annually, more than one million people die from this type of cancer. As a major health concern, the BC incidence is predicted to reach 3.2 million cases per year on a global scale by 2050^[1,2]^. The five-year survival rate varies between 22% and 87%. Factors associated with a worse prognosis include increased axillary lymph node involvement, tumor size >2 cm, presence of comorbidities, lack of surgery, and low socioeconomic status^[3]^. In Iran, the five-year survival rate is unacceptably high (67%), with a mortality rate being twice as high among rural populations^[3,4]^. 

IDC, which originates from milk ducts and invades to the surrounding tissue, is the most common form of BC that accounts for about 80% of all diagnosed BCs. Based on the expression of ER, PR, and HER2 oncogene, IDC breast tumors are classified into five subtypes. Generally, the ER-positive tumors are more prevalent than the ER-negative tumors^[2]^. The ER-positive tumors are smaller and less invasive than ER negative tumors^[5]^. Luminal A and Luminal B are two subtypes of ER/PR-positive tumors. ER-negative tumors consist of three subgroups. One of these subtypes is known as HER2 due to the overexpression of HER2 genes. Basal-like subtype shows the elevated expression of genes typically found in basal cells and normal-like subtype, exhibiting diverse gene expression^[6]^. Identifying the biology of the tumor is highly important and facilitates predicting the prognosis of the disease. Conventionally, tumor size, nodal involvement, distant metastasis, histologic grade, and tumor type were adapted as prognostic and predictive markers^[4,7]^. 

NCAM, known as CD56, is a family member of immunoglobulins that participates in homophilic and heterophilic reactions. All three major isoforms (NCAM-120, NCAM-140, and NCAM-180) are formed by the alternative splicing of a gene on chromosome 11. Naturally, this molecule is expressed in human brain cells and is involved in the production and migration of nerve cells. It is also expressed in natural killer cells, dendritic cells (a group of T lymphocytes, including αβ T and γδ T cells), neuroendocrine cells, and the bone marrow^[8]^. The 140 kDa is the only extraneuronally expressed NCAM isoform. Moreover, 140 kDa isoform was shown to be expressed particularly in a number of highly malignant CD56^+^ neoplasm and was associated with a more aggressive biological behavior and the progression of CD56^+^ precursor lesions of unclear malignant potential^[7,9,10]^. This molecule is used as a marker to diagnose various malignancies such as neural tissue malignancies (e.g. astrocytoma and medulloblastoma), T-cell lymphomas, and neuroendocrine carcinomas^[7]^. The expression of CD56 in malignant tissues is associated with higher rates of invasion, treatment failure, and reduced survival in a wide range of malignancies such as acute lymphoblastic and myeloid leukemia^[7]^.

Recently, a monoclonal antibody directed against the 140 kDa isoform of CD56 was produced in the Shiraz Institute for Cancer Research, Shiraz, Iran^[11]^. In this study, the IHC technique was used to evaluate the expression and prognostic value of CD56 expression (140 kDa isoform) in IDC as a prevalent subtype of BC.

## MATERIALS AND METHODS

Patients and tissue samples

This retrospective study included patients with IDC who had undergone surgical excision of the cancerous mass as the primary treatment at Faghihi Hospital (Shiraz, Iran) between 2009 and 2011. Chemotherapy or radiotherapy had not been performed for these patients before surgery. Data, including age, time of diagnosis, disease subtype, date of the last visit, and date and cause of death in the case of death, were collected. Formalin-fixed and paraffin-embedded tissue blocks were prepared for a total of 65 patients. Samples were selected in such a way to include normal tissue and carcinoma *in situ* in addition to the regions of the invasive tumor. 

Immunohistochemical staining

For IHC staining, formalin-fixed and paraffin-embedded tissue blocks of the patients were cut into 3-µm slices and placed on IHC slides. For deparaffinization and rehydration, the slides were first heated in an oven at 61-62 °C for 15 min and then were immersed in fresh xylene for 30 min. Subsequently, the slides were immersed in pure ethanol for 45 s, followed by 96% ethanol for 30-45 s. Finally, the slides were washed with PBS 1× for 5 min. To reduce false background color, the internal peroxidases were neutralized by adding 200 μl of 10% hydrogen peroxide to each sample in the dark for 3 min. Masked antigens were retrieved by boiling in 250 ml of Tris-EDTA retrieval solution (pH 9) in a pressure cooker for about 20 min. After that, the slides were transferred to a cold water chamber for 20 min before being washed with PBS for 5 min. To prevent non-specific antigen-antibody interactions, samples were covered with 200 μl of blocking solution (10% goat serum in PBS). Following 20 min of incubation, the serum was removed without excessive washing, and 100 μl of primary antibody was added to each slide (Clone 1E3, 1/10 dilution, ICR, Shiraz, Iran)^[11]^ and incubated in a humid chamber for 45 min. Visualization was performed by Master Polymer plus Detection System (Master Diagnostica, Granada, Spain) according to manufacturer’s recommendation. The slides were then dehydrated in graded ethanol solutions, counterstained with hematoxylin and permanently mounted by mounting medium. It should be noted that since the primary antibody was produced against the Pari-ICR cell line, the paraffin-embedded tissue of the patient from whom this cell line was derived was used as the positive control. 

Positive cell quantification

The expression of the CD56 molecule in the cells of the prepared slides (BC tissue, normal tissue, and carcinoma *in situ*) was reported by an experienced pathologist who was blinded to the patients’ data. The reports are based on the DAB chromogen intensity at three different levels: low, moderate, and high expression intensity. Immune cells were excluded from the analysis.

Statistical analysis

All statistical analyses were performed using SPSS software version 16 (IBM, USA). The relationship between CD56 expression and disease parameters was investigated using the Chi-squared test and Fisher’s exact test. To analyze survival, two different periods were taken into account: OS, which is the time from diagnosis to cancer-related death, and DFS, which is the time between the date of surgery and a disease recurrence (local, regional, or distant metastasis). Dissimilarities in survival rates between different groups were assessed using the Kaplan-Meier curves, and significance was assessed using the log-rank test. *p* ≤ 0.05 were considered statistically significant.

## RESULTS

A total of 65 female IDC patients who had received primary therapy with quadrantectomy or radical mastectomy were included in this study. The mean age of the subjects was 48.69 years (ranging 25-79). According to the American Joint Committee on Cancer (AJCC) TNM classification system^[12,13]^, most patients had stage II cancer before surgery. The OS among the subjects was 62.46 months (ranging 8.27-90.17), while the DFS was 54.88 months (ranging 4.30-89.33). The clinicopathologic characteristics of the study subjects are summarized in Table 1.

Expression of the CD56 marker 

**Fig. 1:**
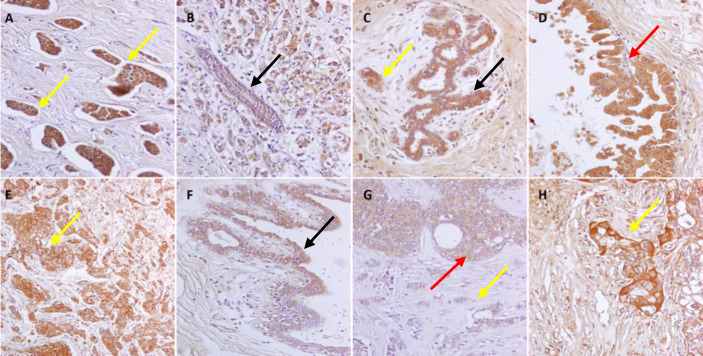
Immunohistochemical staining (×200 magnification) of the IDC tissues for the 140 kDa isoform of the CD56 molecule on positive control (A) and sample tissues (B-H). Black arrows show normal ductal cells with low (B), moderate (C), and high intensity (A and F) of CD56 (140 KDa) expression. Red arrows indicate examples of benign tumor cells with high (D) and moderate (G) CD56 expression intensity. Yellow arrows indicate different staining levels of CD56 including almost negative (G), low (B), moderate (E) and high (A and H) expression.

The patients' tissue samples were stained for the 140 kDa isoform of the CD56 molecule using IHC. The intensity of CD56 expression was classified as low, moderate, or high in cancerous tissue, neighboring normal tissue, and in carcinoma *in situ* (Fig. 1). In two samples, IDC tissues were undetectable, and among the remaining, 12.7% (n = 8) showed low, 25.4% (n = 16) revealed moderate, and 61.9% (n = 39) had high CD56 expression. Cytoplasmic and granular expression patterns were observed. In 18% of cases, carcinoma *in situ* and normal ducts were detectable. Likewise, 11.1% (n = 2) and 38.9% (n = 7) of *in situ* and normal ducts expressed the low level of CD56. Additionally, 33.3% (n = 6) of *in situ* and 11.1% (n = 2) of normal ducts had high expression of CD56. It should be noted that the expression intensity of CD56 in invasive cells was significantly higher than those of *in situ *(*p* = 0.005) and normal cells (*p* = 0.010).The patients were also divided into two or three groups based on different characteristics, and the expression level of CD56 molecule in these groups was compared; the results are summarized in Table 2. Neither the T stage nor the N stage from the TNM classification system had a significant relationship with CD56 expression (*p* = 0.346 and 0.36, respectively; data not shown). As observed in Table 2, only ER expression had a significant, positive relationship with CD56 expression intensity.

**Table 1:** The clinicopathologic characteristics of the study subjects

Characteristic	Status	Frequency (%)	Characteristic	Status	Frequency (%)
**Tumor stage**	I	7 (10.8)	Status at the end of the study	Alive	48 (76.2)
	II	43 (66.2)		Deceased	15 (23.8)
	III	15 (23.1)		Not reported	2 (3.1)
**Histologic grade**	I	12 (18.8)	Post-operative chemotherapy	Yes	39 (95.1)
	II	28 (43.8)		No	2 (4.9)
	III	24 (37.5)		Not reported	24 (36.9)
**Lymph node involvement**	No	30 (47.6)	Post-operative radiotherapy	Yes	30 (76.9)
	Yes	33 (54.4)		No	9 (23.1)
	Not reported	2 (3.1)		Not reported	26 (40)
**Vascular invasion**	No	29 (46.0)	Hormone therapy	Yes	23 (57.5)
	Yes	34 (54.0)		No	17 (42.5)
	Not reported	2 (3.1)		Not reported	25 (38.5)
**Disease recurrence**	Yes	22 (33.8)	HER2 expression	Positive	18 (27.7)
	No	43 (66.2)		Negative	47 (72.3)
**BC type**	HER2+	18 (27.7)	PR expression	Positive	35 (53.8)
	TNBC	18 (27.7)		Negative	30 (46.2)
				
	Luminal	29 (44.6)	ER expression	Positive	36 (55.4)
				Negative	29 (44.6)

TNBC, triple-negative breast cancer

Survival analysis

**Fig. 2:**
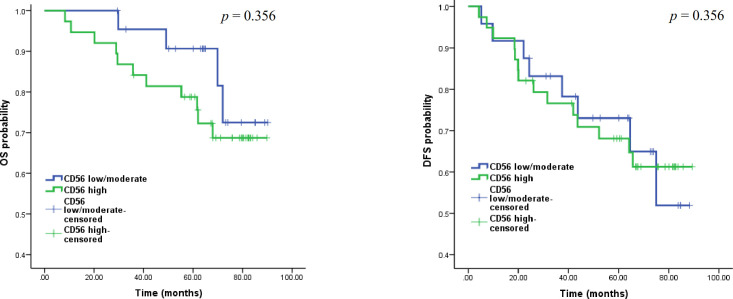
Relationship of CD56 (140 kDa isoform) expression with OS (A) and DFS (B) probability according to the log-rank test and the Kaplan-Meier diagram.

Patients whose primary tumor specimens were used in this study were followed up for up to eight years. During this period, 22 patients experienced disease recurrence and 15 patients died of cancer. In the survival analysis, patients were dichotomized into low/moderate CD56 and high CD56 groups. Log-rank test and the Kaplan-Meier plots were applied to assess the effect of CD56 (140 kDa isoform) expression on the OS and DFS probability of the study subjects. Results showed no significant relationship between the expression of this molecule and both DFS (*p* = 0.976) and OS (0.356) survival times (Fig. 2).

## DISCUSSION

BC is one of the most common malignancies among women, with about a million people dying from the disease every year. To achieve better disease control, we should identify novel diagnostic tools to assist the disease detection in its early stages. Accordingly, specific surface molecules can provide valuable data on the tumor type and disease course. In this study, we evaluated the prognostic value of CD56 expression in IDC by assessing its relationship with a wide range of clinicopathologic characteristics. The expression of this marker was also compared between invasive tumor cells, benign tumor cells, and normal ducts. 

Prior studies have examined the expression of the CD56 molecule in two ways, namely its diagnostic/prognostic role, as well as its usefulness in the development of antibodies that target cancerous tissue. In the present study, the expression of the CD56 molecule was found to be independent of survival (both overall and disease-free survival), recurrence, TNM stage, lymphatic involvement, and vascular invasion. In contrast, Aref *et al.*^[14]^ found the expression of this molecule to be associated with decreased survival among lymphoblastic leukemia patients. Aloysius *et al.*^[15]^ reported a correlation between CD56 expression and perineural invasion in periampullary cancer, with the latter comprising an independent predictor of poor survival. Furthermore, CD56 140 kDa expression predicts a more aggressive behavior in several malignant tumors^[10,14-17]^. These results are in contrast with our findings and might be due to the differences between antibody isoforms in our study and the studies mentioned above. Another notable finding of the present study was that IDC tissue, carcinoma *in situ*, and normal cells could not be differentiated based on CD56 expression because of significance association between the mentioned cells in terms of CD56 expression intensity. Contrasting with our results, CD56 marker is effective in differentiating between papillary thyroid carcinoma and benign thyroid lesions^[18,19]^.

**Table 2:** Relationship between CD56 expression in BC (IDC) tissues and clinicopathological parameters

**Variable**	**CD56 expression intensity**			***p*** ** value**
	**Low**	**Moderate**	**High**	
Age				
>48 years	3 (4.8)	4 (6.3)	24 (38.1)	0.340
<48 years	5 (7.9)	12 (19.0)	15 (23.8)	
Cancer-caused dearh				
Yes	7 (11.5)	12 (19.7)	27 (44.3)	0.753
No	1 (1.6)	3 (4.9)	11 (18)	
				
Disease recurrence				
Yes	6 (9.5)	10 (15.9)	25 (39.7)	0.861
No	2 (3.2)	6 (9.5)	14 (22.2)	
				
TNM stage				
I	2 (3.2)	2 (3.2)	3 (4.8)	0.373
II	4 (6.3)	9 (14.3)	29 (46.0)	
III	2 (3.2)	5 (7.9)	7 (11.1)	
				
Histological grade				
I	1 (1.6)	1 (1.6)	10 (16.1)	0.320
II	2 (3.2)	7 (11.3)	17 (27.4)	
III	5 (8.1)	7 (11.3)	12 (19.4)	
				
Lymph node involvement				
No	5 (8.2)	7 (11.5)	18 (29.5)	0.712
Yes	3 (4.9)	9 (14.8)	19 (31.1)	
				
Vascular invasion				
Yes	1 (1.6)	9 (14.8)	19 (31.1)	0.128
No	6 (9.8)	6 (9.8)	20 (32.8)	
No	2 (3.2)	6 (9.5)	14 (22.2)	
				
BC type				
HER2+	2 (3.2)	3 (4.8)	12 (19)	0.057
TNBC	4 (6.3)	8 (12.7)	6 (9.5)	
Luminal	2 (3.2)	5 (7.9)	21 (33.3)	
				
HER2 expression				
Yes	2 (3.2)	3 (4.8)	12 (19)	0.775
No	6 (9.5)	13 (20.6)	27 (42.9)	
				
PR expression				
Yes	3 (4.8)	4 (6.3)	22 (34.9)	0.090
No	5 (7.9)	12 (19.0)	17 (27.0)	
				
ER expression				
Yes	3 (4.8)	5 (7.9)	27 (42.9)	0.023
No	5 (7.9)	11 (17.5)	12 (19.0)	

Values are expressed as frequency (percentage). TNBC, triple-negative breast cancer

The expression of surface molecules in cancer cells, in addition to being a potential diagnostic and prognostic tool, can be used to treat the disease. For instance, lorvotuzumab mertansine is an antibody that targets the CD56 molecule. This antibody can bind to CD56-positive cells and stimulate an immune response. In one study, Socinski *et al.*^[20]^ divided patients with small cell lung cancer into two groups, one provided with only standard treatment including carboplatin/etoposide and the other receiving lorvotuzumab mertansine besides standard treatment. The authors reported that the anti-CD56 agent not only failed to improve the effectiveness of treatment but also increased the incidence of side effects like drug poisoning and serious infections^[20]^. Although anti-CD56 treatment was not observed to be effective in the treatment of small cell lung cancer, its role in BC treatment is yet to be studied. In this study, we observed that the expression of CD56 molecule (140 kDa isoform) in IDC tissue had a significant relationship with ER expression. In fact, 42.9% of ER+ cases possessed high-intensity CD56 expression, compared with roughly 19% in the ER- group. The ER is found mostly in uterus, ovaries, kidneys, breasts, bones, and cardiovascular tissues as well as nervous systems. This protein receptor is involved in cell replication, and differentiation; it is found mainly in the cell nucleus but can also be expressed in the cytoplasm, mitochondria, and at the cell surface. Although ER is expressed in less than 10% of normal breast tissue, its expression rate reaches 50-80% in BC cells. In ER+ patients, antihormonal therapy such as tamoxifen and raloxifene are commonly used. The expression of ER molecules at the level of BC cells is associated with the disease prognosis, such that ER+ patients generally have a better prognosis than ER- patients^[21,22]^. Hence, in line with our findings, it can be concluded that the expression of the CD56 molecule is indirectly related to the disease prognosis among IDC patients. Nonetheless, we found no significant relationship between CD56 expression and overall or disease-free survival. 

The significance of the 140 kDa isoform of CD56 has been investigated in several studies^[7,9,10]^. Our data indicated the CD56 marker offered no prognostic value in terms of predicting the OS or DFS for up to eight years after primary surgery for IDC. Furthermore, the intensity of its expression was similar between normal, non-invasive, and invasive cells. Among the studied clinicopathologic parameters, only ER expression had a significant relationship with CD56 expression intensity in BC tissue. Considering the fact that ER+ tumors are associated with better outcome than ER- tumors, the CD56 marker may be indirectly associated with a more favorable prognosis among IDC patients. Broader studies with larger sample sizes seem to be warranted.

## DECLARATIONS

### Ethical statement

The above-mentioned sampling protocols were approved by the Research Ethics Committee of the Shiraz University of Medical Sciences, Shiraz, Iran (ethical code: IR.SUMS.MED.REC.1399.193). Written informed consents were provided by all patients. 

### Data availability

Data supporting this article are included within the article and supplementary file. 

### Author contributions

KN: collected the tissue samples, patient information and prepared the first draft of the manuscript; AS: analyzed all immunohistochemical analysis and contributed to manuscript editing; AG: was the principal investigator of the project and edited the final version of the manuscript. 

### Conflict of interest

None declared.

### Funding/support

This article was extracted from the dissertation of Kianoush Niknam completed as a part of the requirements of the MD degree and funded by the Vice Chancellery for Research of the Medical School of Shiraz University of Medical Sciences (Project No. 20744 dated 27/11/2019). 
